# The Characteristics of Action Potentials in Primo Vessels and the Effects of Acetylcholine Injection to the Action Potentials

**DOI:** 10.1155/2013/657969

**Published:** 2013-06-04

**Authors:** Seong Jin Cho, Jaekwan Lim, Sun Hee Yeon, O. Sang Kwon, Kwang-Ho Choi, Sun-Mi Choi, Yeon-Hee Ryu

**Affiliations:** ^1^Acupuncture, Moxibustion, and Meridian Research Group, Medical Research Division, Korea Institute of Oriental Medicine, Daejeon 305-811, Republic of Korea; ^2^Advanced Institutes of Convergence Technology, Seoul National University, Suwon, Republic of Korea

## Abstract

In a previous study, we found that Primo vessels generate different action potentials in smooth muscles, but this study compared the pulse shape to distinguish the two tissues. Thus, a more sophisticated extracellular experiment was performed in this study using an acetylcholine injection; we then observed changes in the amplitude, FWHM (full width at half maximum), and period to explore Primo vessel function. A third type of pulse was recorded for Primo vessels. We observed fast depolarizing and repolarizing phases for this pulse. Further, its FWHM was 30 ms between smooth muscles and neurons. Acetylcholine affected only the period. The amplitude and FWHM were consistent after injection. Primo-vessels generated action potentials at twice the frequency after injection. From the results, we speculate that Primo-vessels perform a role in transferring signals in a different manner, which may be relevant for acupuncture treatment.

## 1. Introduction

Over the last decade Primo vascular system has attracted interests among researchers both anatomically and histologically. However, it is still unclear what functions Primo tissues do. Some researchers have found Primo tissues in organ surface, lymphatic vessels, cardiac vascular vessels, and brain. These findings have led them to consider Primo vascular system another circulating system [1–4]. Moreover, Soh claimed that Primo nodes and Primo-vessels were related to acupuncture points and Primo vascular system might be an extension of meridians [5]. Park started to measure action potentials in Primo-node for the first time in 2000s. He measured resting and spontaneous potentials and observed the affection of drugs [6]. More researchers have studied Primo-vessel electrophysiological characteristics after this study, and they have found bioelectrical signals from Primo-vessels and lymphatic vessels were different. However, the results have not been sufficient to clarify the functions [7, 8]. In our previous study on Primo-vessel action potentials, we found that two types of pulses were generated that differed from the pacemaker pulse for smooth muscles in the small intestine. This result indicates that Primo-vessels have a distinct function from smooth muscles. However, it was difficult to determine what role Primo-vessels played because we only compared pulse shape to distinguish Primo-vessels and smooth muscles [9]. The action potentials from the different tissues have distinct pulse components, such as amplitude, duration, and frequency. Further, neurotransmitters can affect these components [10–13]. Thus, the pulse components must be analyzed, and the reaction of tissues to neurotransmitters must be measured. In this study, we analyzed the action potentials from Primo-vessels and observed the effects of acetylcholine on the pulse component.

## 2. Materials and Methods

### 2.1. Animals and Tissue Preparation

Male 7-week-old Sprague-Dawley rats weighing 250–320 g were used. The rats were anesthetized with a 1.5 g/kg urethane (C_3_H_7_NO_2_) injection into the femoral region, and the entire surgical procedure was performed with the anesthetized rat. A midline abdominal incision was generated, and the internal organs were exposed. The large intestine surface Primo-vessels were removed from the rats and placed on a Sylgard. We then added phosphate buffered saline (PBS, which contained (in mM) 137 NaCl, 27 KCl, 10 Na_2_HPO_4_, and 2 KH_2_PO_4_) to the sample.

### 2.2. Equipment

The tissue preparations were viewed under a microscope (SMZ1500, Nikon, Japan), and a Fiber-Lite supplied the light (MI-150, Dolan Jenner industries, MA, USA). A second microscope (SZ61, Olympus, Japan) and micromanipulator (MP-225, Sutter Instrument, CA, USA) were used to insert the electrode (TM33B01, World Precision Instruments, FL, USA) into the tissue. An Ag/AgCl electrode (EP1, World Precision Instruments, FL, USA) was used as the reference electrode. The electric signals were amplified by a primary amplifier that contained a head amplifier (Duo773, World Precision Instruments, FL, USA) and acquired by using a data acquisition system (PowerLab/16SP, ADInstruments, CO, USA). A data acquisition program (LabChart 6, ADInstruments, CO, USA) was used to record the data, and the data were analyzed using Microsoft Excel 2010 (Microsoft, WA, USA).

### 2.3. Extracellular Recording

An electrode was placed in the tissue on the Sylgard. The tissue was perfused with Krebs' solution at an approximate 5 mL/min constant flow rate. The Krebs' solution contained (in mM) 10.10 D-glucose, 115.48 NaCl, 21.90 NaHCO_3_, 4.61 KCl, 1.14 NaH_2_PO_4_, 2.50 CaCl_2_, and 1.16 MgSO_4_. This solution was at pH 7.4 and 36°C. The solution temperature in the organ bath was maintained at 36~38°C. The electrical responses were amplified, low pass filtered (50 Hz), and recorded (time interval: 0.0005 sec) on a computer. The laboratory was isolated from electromagnetic noise by a Faraday cage. Acetylcholine (A6625-25G, Sigma Aldrich, MO, USA) was diluted 1000 times and injected into the solution 5 minutes after the action potential was detected. After recording the action potentials, H&E staining was applied to the tissue.

### 2.4. Analysis

Two sections, 1 minute before and 1 minute after acetylcholine injection, were extracted in all samples. The amplitude, FWHM (full width at half maximum), and period, the time difference between one peak and the next peak, were calculated from the action potentials and averaged totally in each and compared by performing *t*-test.

## 3. Results

**Figure 1:**
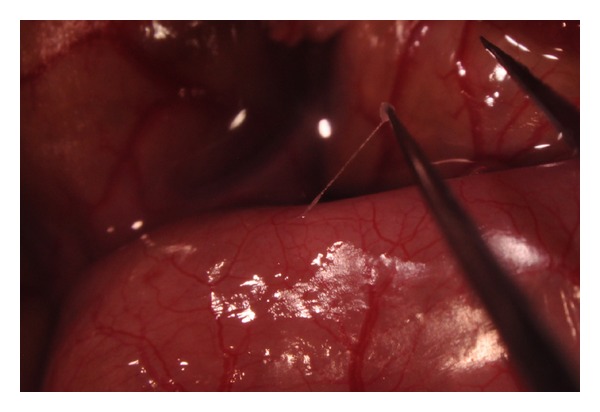
Stereomicroscopic image of Primo-vessel used in this study. This sample was taken from large intestine in SD rat. This sample had thread-like structure and milky-white color as the other studies reported.

**Figure 2:**
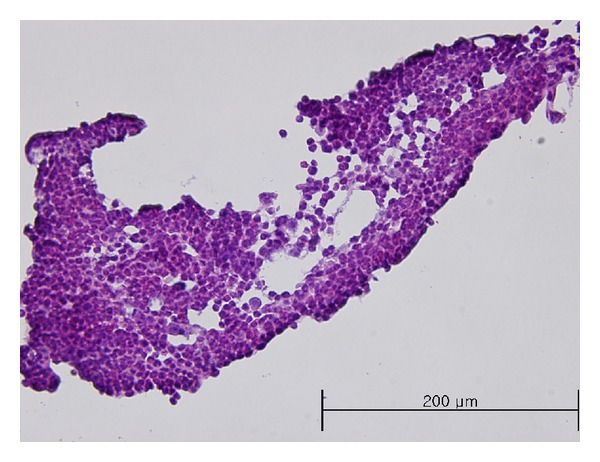
H&E staining result of the sample shown Figure 1. It was the same histological characteristic of Primo-vessels with other studies that the tissue was filled with cells.

We tried to measure action potentials in 10 samples, and these samples were verified by comparing anatomical and histological characteristics with other studies.  Figure 1 shows the stereomicroscopic image of a sample used in this study, and Figure 2 shows the H&E staining result of the sample. The anatomical structure and the histological feature were same with other studies [14, 15].

**Figure 3:**
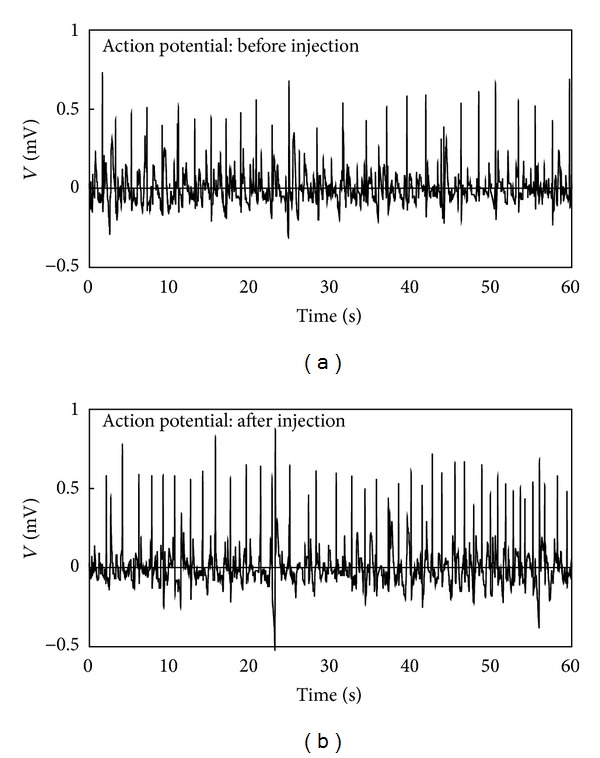
Representative Primo-vessel action potentials. (a) The action potentials for a section 1 minute before acetylcholine injection. (b) The action potentials for a section 1 minute after acetylcholine injection. The pulses rose and fell rapidly and were generated periodically in both sections.

 We measured spontaneous action potentials before and after acetylcholine injection in 3 samples.  Figure 3 shows the representative action potentials generated from Primo-vessels in this study. The pulses had rapid depolarizing and repolarizing phases, such as the Type I pulse in our previous study. However, the difference from our previous study is that the action potentials were generated periodically both before and after acetylcholine injection.

**Figure 4:**
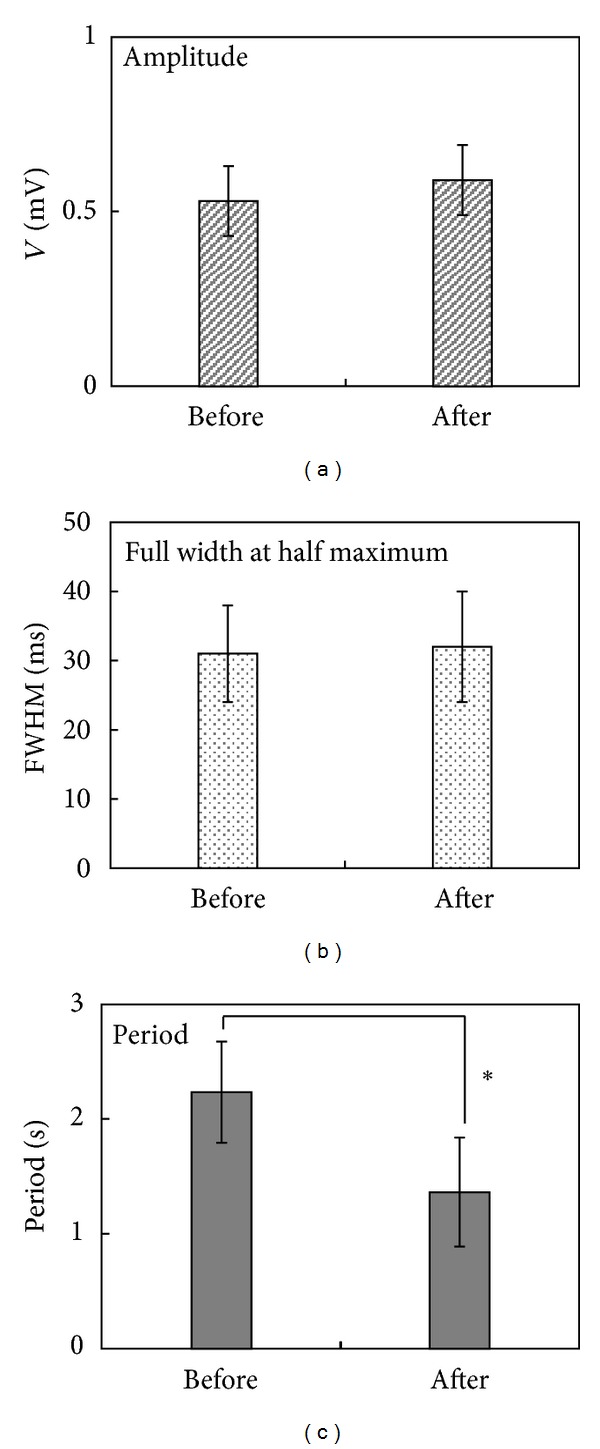
A comparison of the amplitude, FWHM, and period for sections before and after acetylcholine injection. (a) The amplitude was slightly but not significantly increased after injection. (b) The FWHM was maintained in both sections. (c) The period decreased after injection. Acetylcholine activated the Primo-vessels.

For a detailed comparison of the action potentials from the before and after sections, the amplitude, FWHM, and period were calculated in each section. The pulses in before section had a 0.53 ± 0.10 mV mean amplitude, and the pulses in after section had a 0.59 ± 0.10 mV mean amplitude. The amplitude was slightly but not significantly increased after injection. The FWHM for the pulses was around 30 ms for both sections. However, there was a significant variation in the period. The Primo-vessels generated an action potential with a 2.234 ± 0.442 s period before injection. After injection, the period decreased by half, 1.364 ± 0.474 (Table 1).  Figure 4 shows a comparison of the amplitude, FWHM, and period for the before and after injection sections in all samples.

**Figure 5:**
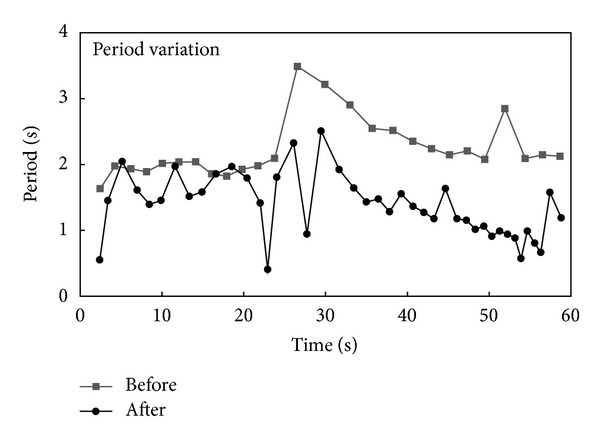
Representative time variation for period before and after injection. Under normal conditions, the pulses generated with a 2 s period. After acetylcholine injection, the period decreased gradually to half of the pre-injection value.

Further, we observed time variations for the period.  Figure 5 shows the variation in the period for the time domain. This figure was gained from the same sample which was used in Figure 3. Primo-vessels generated action potentials with a 2 s period before acetylcholine injection, but the period decreased gradually to 1 s.

## 4. Discussion

In this study, we analyzed action potential pulse components. Because of inaccuracy in the extracellular recording amplitudes, the amplitude absolute values had no meaning. However, we did observe an effect from acetylcholine injection by comparing the amplitude change before and after injection. The action potential amplitudes for Primo-vessels were slightly increased by approximately 13% after the acetylcholine injection. It is hard to assert that acetylcholine facilitates action potentials in Primo-vessels with larger amplitudes because the amplitude variation is in the error range. Previous studies have reported the same results; that is, acetylcholine had no effect on action potential amplitude. The pulses had approximately 30 ms FWHMs in this study, which is approximately one-tenth of the value reported in our previous study. These pulses do not belong to Type I or Type II. Thus, we categorized these pulses as Type III with fast depolarizing and repolarizing phases. The FWHM for these pulses was shorter than in smooth muscles from the small intestine or lymphatic vessels (~500 ms) and longer than in neurons (~1 ms) [16]. The small intestine and lymphatic vessels generate an action potential to transfer materials. Neuron spikes are generated when neurons exchange electrical signals [17]. Primo-vessel has median values of its size and FWHM between smooth muscle and a neuron. This result indicates that Primo-vessels perform different functions in smooth muscles and neurons. We speculate that Primo-vessels transfer signals in distinct manners for neurons and do not directly move materials, such as through the small intestine and lymphatic vessels. Primo-vessels were considered the substances in acupuncture points and meridians [5]. Therefore, signals may contain information from acupuncture stimulation if Primo-vessels have such a role. The FWHM was maintained after the acetylcholine injection. Thus, acetylcholine did not affect the FWHM for the action potentials in Primo-vessels. This result is consistent with other studies involving acetylcholine.

 However, there was a significant change in the period after acetylcholine injection. The period is the time interval between the two serial pulses. Primo-vessels generated Type III pulses with a 2.234 s period on average under normal condition. Acetylcholine decreased the period to 1.364 s. Thus, acetylcholine enhanced Primo-vessels activation and generated action potentials more frequently. In Figure 5, the period was maintained as under normal conditions for 30 s and then gradually decreased to half the value of before section.

## 5. Conclusion

We found that Primo-vessels generated a third type of pulse with fast depolarization and repolarization. This type had a uniform amplitude, 30 ms FWHM, and 2 s period in normal condition. Primo-vessels' function was considered transferring electrical signals rather than carrying material due to the very short FWHM. Further, the amplitude and the FWHM of the action potentials from Primo-vessels were not changed by acetylcholine. However, acetylcholine decreased the period of the action potentials to 1 s, half value of normal condition. It means that Primo vascular system can be controlled by acetylcholine. Further studies are necessary to clarify the functions of Primo-vessels and to establish the relation between Primo vascular system and meridian system.

## Figures and Tables

**Table 1:** The amplitude, FWHM and period for the Primo-vessel action potentials. The amplitude is the difference between the maximum and rest potentials. The FWHM is the time difference between two points that is equal to half of the amplitude. The period is the time difference between one peak and the next peak. The amplitude was slightly but not significantly increased after acetylcholine injection. The FWHM was not changed by acetylcholine. The period was decreased after injection.

	Amplitude (mV)	FWHM (ms)	Period (s)
Before injection	0.53 ± 0.10	31 ± 7	2.234 ± 0.442
After injection	0.59 ± 0.10	32 ± 8	1.364 ± 0.474
